# 418. Fosfomycin Dosing Regimens for The Treatment of Carbapenem-Resistant *Enterobacteriaceae* Infections in Patients Receiving Continuous Renal Replacement Therapy: a Monte Carlo Simulation

**DOI:** 10.1093/ofid/ofac492.495

**Published:** 2022-12-15

**Authors:** Sukrit Kanchanasurakit, Chansinee Srisawat, Charles E McPherson, Weerayuth Saelim, Wuttikorn Siriplabpla, Pornsinee Suthumpoung, Wichai Santimaleeworagun

**Affiliations:** Division of Clinical Pharmacy, Department of Pharmaceutical Care, School of Pharmaceutical Sciences, University of Phayao, Phayao, Thailand, Muang Phrae, Phrae, Thailand; Division of Clinical Pharmacy, Department of Pharmaceutical Care, School of Pharmaceutical Sciences, University of Phayao, Phayao, Thailand, Muang Phrae, Phrae, Thailand; Department of Pharmacy Practice, University of Illinois at Chicago College of Pharmacy, Chicago, Illinois, USA, Chicago, Illinois; Department of Pharmacy, Faculty of Pharmacy, Silpakorn University, Nakhon Pathom, Thailand, Muang Nakhon Pathom, Nakhon Pathom, Thailand; Division of Nephrology, Department of Medicine, Phrae Hospital, Phrae, Thailand, Muang Phrae, Phrae, Thailand; Division of Clinical Pharmacy, Department of Pharmaceutical Care, School of Pharmaceutical Sciences, University of Phayao, Phayao, Thailand, Muang Phrae, Phrae, Thailand; Faculty of Pharmacy, Silpakorn University - Muang, Nakorn Pathom Thailand, Muang, Nakhon Pathom, Thailand

## Abstract

**Background:**

To predict the appropriate dosing of intravenous fosfomycin for treatment of carbapenem-resistant Enterobacteriaceae (CRE) infection in continuous renal replacement therapy (CRRT) patients.

**Methods:**

Minimum inhibitory concentration (MIC) values of all isolates were determined by E-test method. Population pharmacokinetic parameters were obtained from a previously published study. The percentages of a 24-hour period in which the drug concentration exceeded the MIC (%T >MIC) were defined to be 70% T >MIC and 100% T >MIC, respectively. In addition, the 24-hour area under the unbound concentration-time curve over the MIC (AUC_0-24_/MIC) of 45 mg·h/L was used as a target value. All dosing regimens were estimated for the probability of target attainment (PTA) using a Monte Carlo simulation.

**Results:**

For the effluent rate of 20 mL/kg/h, the PTA for reaching 70% T >MIC, 100% T >MIC, and AUC_0-24_/MIC of 45 mg·h/L was achieved in pathogens with a MIC of 24 mg/L, 12 mg/L, and 24 mg/L in all regimens, respectively. Meanwhile for the effluent rate of 25 mL/kg/h, the PTA for reaching 70% T >MIC, 100% T >MIC, and AUC_0-24_/MIC of 45 mg·h/L was achieved in organisms with a MIC of 16 mg/L, 12 mg/L and 24 mg/L in all regimens, respectively.

**Conclusion:**

The appropriate fosfomycin dosing regimens for CRE infections in critically ill patients receiving CRRT were suggested based on pharmacokinetic/pharmacodynamic targets, MIC values, and effluent rates. Clinical validation is warranted.

**Disclosures:**

**All Authors**: No reported disclosures.

